# Features and risk factors of carotid atherosclerosis in a population with high stroke incidence in China

**DOI:** 10.18632/oncotarget.15415

**Published:** 2017-02-16

**Authors:** Yanqiu Zhang, Lingling Bai, Min Shi, Hongyan Lu, Yanan Wu, Jun Tu, Jingxian Ni, Jinghua Wang, Li Cao, Ping Lei, Xianjia Ning

**Affiliations:** ^1^ Department of Neurology, Tianjin Nankai Hospital, Tianjin, China; ^2^ Department of Neurology, Tianjin Medical University General Hospital, Tianjin, China; ^3^ Department of Epidemiology, Tianjin Neurological Institute, Tianjin, China; ^4^ Tianjin Neurological Institute, Key Laboratory of Post-Neuroinjury Neuro-Repair and Regeneration in Central Nervous System, Ministry of Education and Tianjin City, Tianjin, China; ^5^ Center of Clinical Epidemiology, Tianjin Medical University General Hospital, Tianjin, China; ^6^ Department of Geriatrics, Tianjin Medical University General Hospital, Tianjin, China

**Keywords:** carotid intima-media thickness, carotid plaque, atherosclerosis, risk factors, epidemiology

## Abstract

Epidemiological studies have reported associations between traditional cardiovascular risk factors and carotid intima-media thickness (CIMT) or carotid plaque. However, definite risk factors at different phases of carotid atherosclerosis remain controversial. We aimed to explore risk factors and characteristics of carotid atherosclerosis at different stages in a low-income population with a high incidence of stroke in China. Between April 2014 and January 2015, we recruited 3789 stroke-free and cardiovascular disease-free residents aged ≥ 45 years. B-mode ultrasonography was performed to measure CIMT and the presence of carotid plaque. Traditional risk factors were compared between the increased CIMT group and normal CIMT group, and between those with and without carotid plaque. A total of 3789 participants were assessed in this study, with a mean age (standard deviation) of 59.92 (9.70) years. The prevalence of increased CIMT and carotid plaque increased with older age and higher education levels. Age, hypertension, diabetes, and high low-density lipoprotein cholesterol levels were risk factors for increased CIMT and carotid plaque. Furthermore, compared to never smoking, passive smoking was positively associated with increased CIMT, with an odds ratio (95% confidence interval) of 1.26 (1.05, 1.53; *P* = 0.016); high body mass index was an obvious protective factor against carotid plaque, with an odds ratio (95% confidence interval) of 0.97 (0.95, 0.99; *P* = 0.004). It is important to identify factors associated with atherosclerosis to prevent cardiovascular disease and stroke and reduce the burden of stroke in this high-risk population.

## INTRODUCTION

Although there was no significant change in the age-standardized incidence of stroke in low-income and middle-income countries between 1990 and 2010, there was a significant reduction in mortality rates both in high-income countries and in low- and middle-income countries [1]. According to 2010 statistics, stroke was the leading cause of death in rural areas and the third leading cause of death in urban areas in China [2]. Our previous studies demonstrated that the age-standardized incidence of first-ever stroke dramatically increased over the past two decades [3–5]. The prevalence of conventional risk factors was high and significantly increased during the period from 1991 to 2011 in this population [6, 7].

Carotid artery atherosclerosis (AS) is a strong predictor for cardiovascular disease (CVD) and ischemic stroke events as a result of both luminal stenosis and plaque rupture [8–12]. Carotid intima-media thickness (CIMT), carotid plaque, and carotid stenosis are markers for carotid AS, and they can reflect different phases of carotid artery AS. Increased CIMT mainly represents the early phase of carotid AS, whereas the presence of carotid plaque and carotid stenosis reflects later and advanced phases of the atherosclerotic process [13–15]. Moreover, carotid plaque was found to be a stronger predictor of stroke risk than stenosis [16]. Epidemiological studies have reported associations between traditional cardiovascular risk factors and CIMT or carotid plaque [11, 17–19]. However, factors related to CIMT and carotid plaque are unknown in China, especially among low-income populations.

Therefore, in this study, we aimed to explore the risk factors and characteristics of carotid AS at different phases among a low-income population in China with a high incidence of stroke.

## RESULTS

### Demographics, lifestyle, and risk factors of the study population

A total of 3789 residents (mean age, 59.92 years) were assessed in this study. There were more women than men (58.8% *vs*. 41.2%), with mean ages of 61.13 years in men and 59.07 years in women. Men were more likely to be older and have higher educational levels; men also exhibited higher prevalence rates of hypertension, diabetes, current smoking, and alcohol consumption. Moreover, systolic blood pressure (SBP) and diastolic blood pressure (DBP) levels were greater in men than in women, but the levels of total cholesterol (TC), triglycerides (TG), high-density lipoprotein cholesterol (HDL-C), and low-density lipoprotein cholesterol (LDL-C) were greater in women than in men (all *P* < 0.0001; Table 1).

**Table 1:** Demographic characteristics of all subjects in this study

Risk factors	Total	Men	Women	*P*
Total:	3789 (100)	1560 (41.2)	2229 (58.8)	
Age, mean(SD), years	59.92 (9.70)	61.13 (9.90)	59.07 (9.47)	<0.0001
Age group, *n* (%)				<0.0001
45∼54 years	1236 (32.6)	430 (27.6)	806 (35.2)	
55∼64 years	1514 (40.0)	632 (40.5)	882 (39.6)	
65∼74 years	724 (19.1)	338 (21.7)	386 (17.3)	
≥75 years	315 (8.3)	160 (10.3)	155 (7.0)	
Education, mean(SD), years	5.48 (6.54)	6.40 (3.22)	4.84 (3.61)	<0.0001
Education, *n* (%)				<0.0001
0 years	659 (17.4)	137 (8.8)	522 (23.4)	
1∼6 years	1694 (44.7)	699 (44.8)	995 (44.6)	
> 6 years	1436 (37.9)	724 (46.4)	712 (31.9)	
Smoking status, *n* (%)				<0.0001
Never smoking	2840 (75.0)	664 (42.6)	2176 (97.6)	
Ever smoking	173 (4.6)	166 (10.6)	7 (0.3)	
Current smoking	776 (20.4)	730 (46.8)	46 (2.1)	
Alcohol consumption, *n* (%)				<0.0001
Never drinking	3198 (84.4)	999 (64.0)	2199 (98.7)	
Ever drinking	49 (1.3)	48 (3.1)	1 (0.0)	
Current drinking	542 (14.3)	513 (32.9)	29 (1.3)	
Hypertension, *n* (%)	2583 (68.2)	1111 (71.2)	1472 (66.0)	0.001
Diabetes, *n* (%)	533 (14.1)	216 (14.1)	317 (14.5)	0.719
Obesity, *n* (%)	888 (23.4)	323 (20.7)	565 (25.3)	0.001
SBP, mean(SD), mmHg	146.42 (22.17)	147.76 (21.41)	145.49 (22.64)	0.002
DBP, mean(SD), mmHg	86.81 (11.40)	88.50 (11.22)	85.62 (11.39)	<0.0001
BMI, mean(SD), Kg/m^2^	25.57 (3.68)	25.20 (3.44)	25.82 (3.82)	<0.0001
FBG, mean(SD), mmol/L	5.92 (1.57)	5.91 (1.42)	5.93 (1.67)	0.660
TC, mean(SD), mmol/L	4.87 (1.09)	4.62 (1.00)	5.04 (1.11)	<0.0001
TG, mean(SD), mmol/L	1.76 (1.24)	1.61 (1.24)	1.87 (1.22)	<0.0001
HDL-C, mean(SD), mmol/L	1.46 (0.46)	1.39 (0.43)	1.50 (0.48)	<0.0001
LDL-C, mean(SD), mmol/L	2.70 (1.25)	2.61 (1.20)	2.76 (1.28)	<0.0001

Abbreviations: SD, standard deviation; SBP, systolic blood pressure; DBP, diastolic blood pressure; BMI, body mass index; FBG, fasting blood glucose; TC, total cholesterol; TG, triglycerides; HDL-C, high density lipoprotein cholesterol; LDL-C, low density lipoprotein cholesterol.

### Prevalence of increased CIMT and carotid plaque by demographics, lifestyle, and risk factors

Table 2 shows that the frequency of increased CIMT tended to be greater in men, older participants, those with higher educational levels, current smokers, those who consumed alcohol, and those with hypertension and diabetes (*P* < 0.001). In the increased CIMT group, TC and LDL-C levels were greater, but TG levels were lower. Similar to increased CIMT, a high prevalence of carotid plaque was found in men, older participants, and those with current smoking status, hypertension, and diabetes; the prevalence of carotid plaque decreased with higher educational levels (*P* < 0.001). TC and LDL-C levels were higher in the carotid plaque group, but a reverse trend was found with body mass index (BMI).

**Figure 1:**
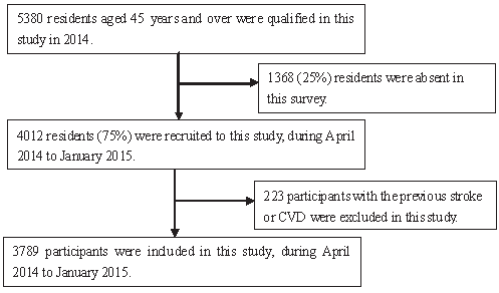
Flow chart of participants

**Table 2:** The prevalence of carotid plaque and IMT increased by demographical characteristics and risk factors*

Risk factors	IMT increased		CP	
	Value	*P*	Value	*P*
Total:	957 (25.3)	< 0.001	1574 (41.5)	<0.001
Men	515 (33.0)		782 (50.1)	
Women	442 (19.8)		792 (35.5)	
Age group, n (%)		< 0.001		<0.001
45∼54 years	158 (12.8)		281 (22.7)	
55∼64 years	384 (25.4)		684 (45.2)	
65∼74 years	276 (38.1)		390 (53.9)	
≥75 years	139 (44.1)		219 (69.5)	
Education:		< 0.001		<0.001
0 years	207 (31.4)		318 (48.3)	
1∼6 years	468 (27.6)		759 (44.8)	
> 6 years	282 (19.6)		497 (34.6)	
Smoking status:		< 0.001		<0.001
Never smoking	493 (24.3)		808 (41.6)	
Passive smoking	253 (22.5)		402 (35.8)	
Ever smoking	40 (27.4)		66 (45.2)	
Current smoking	19 (33.1)		298 (51.6)	
Alcohol consumption:		< 0.001		0.255
Never drinking	765 (23.9)		1311 (41.0)	
Ever drinking	18 (36.7)		31 (63.3)	
Current drinking	174 (32.1)		232 (42.8)	
Hypertension		< 0.001		<0.001
Yes	772 (29.9)		1213 (47.0)	
No	185 (15.3)		361 (29.9)	
Diabetes		< 0.001		<0.001
Yes	171 (32.1)		282 (52.9)	
No	765 (24.0)		1263 (39.6)	
BMI		0.436		0.002
With IMT increased/CP	25.49 (3.63)		25.35 (3.70)	
Without IMT increased/CP	25.59 (3.70)		25.72 (3.67)	
TC		0.038		<0.001
With IMT increased/CP	4.93 (1.05)		4.99 (1.15)	
Without IMT increased/CP	4.84 (1.10)		4.78 (1.04)	
TG		0.038		0.903
With IMT increased/CP	1.69 (1.36)		1.76 (1.13)	
Without IMT increased/CP	1.78 (1.19)		1.76 (1.31)	
HDL-C		0.066		0.582
With IMT increased/CP	1.43 (0.44)		1.45 (0.45)	
Without IMT increased/CP	1.46 (0.47)		1.46 (0.47)	
LDL-C		< 0.001		<0.001
With IMT increased/CP	2.88 (1.24)		3.07 (1.44)	
Without IMT increased/CP	2.63 (1.25)		2.43 (1.02)	

*all data was presented as rate (%) with standard error of rate.

TC, total cholesterol; TG, triglycerides; HDL-C, high density lipoprotein cholesterol; LDL-C, low density lipoprotein cholesterol.

### Carotid plaque load by CIMT group

The prevalence rates of 1, 2, and ≥3 plaques among participants were 58.2%, 30.5%, and 11.3%, respectively. The corresponding mean total plaque areas were 11.44 mm^2^, 28.63 mm^2^, and 62.70 mm^2^, respectively. In the increased CIMT group, the frequency of ≥ 3 plaques was 16.7%, and the mean total plaque area was 68.77 mm^2^ (Table 3).

**Table 3:** Description of the load with presence carotid plaques by numbers and total areas of plaques in this population

Characteristics	Presence 1 plaque	Presence 2 plaques	Presence ≥3 plaques
Total:			
Cases, *n* (%)	916 (58.2)	480 (30.5)	178 (11.3)
Total plaque areas, mm^2^	11.44 (7.96)	28.63 (15.73)	62.70 (36.42)
Without increased CIMT:			
Cases, *n* (%)	642 (63.4)	286 (28.3)	84 (8.2)
Total plaque areas, mm^2^	10.87 (6.94)	26.88 (15.18)	55.91 (26.57)
With increased CIMT:			
Cases, *n* (%)	274 (48.8)	194 (34.5)	94 (16.7)
Total plaque areas, mm^2^	12.77 (9.85)	31.21 (16.21)	68.77 (42.60)

### Risk factors of increased CIMT and carotid plaque

The multivariate analysis results indicated that male sex, older age, lower education level, hypertension, diabetes, passive smoking, and high LDL-C level were independent risk factors of increased CIMT. The odd ratios with 95% confidence intervals (ORs [95% CIs]) were 1.94 (1.59, 2.37) for men compared to women; 1.94 (1.55, 2.41) for age 55-64 years, 3.22 (2.49, 4.16) for age 65-74 years, and 4.03 (2.91, 5.57) for age ≥ 75 years compared to age 45-54 years; 1.85 (1.52, 2.24) for hypertension compared to non-hypertension; 1.26 (1.02, 1.55) for diabetes compared to non-diabetes; 1.26 (1.05, 1.53) for passive smoking compared to never smoking, and 1.15 (1.08, 1.22) for LDL-C level increased by 1 mmol/L (Table 4).

**Table 4:** Multivariate analysis for the risk factors of increased CIMT in this study

Risk factors	References	OR (95% CI)	*P*
Men	Women	1.94 (1.59, 2.37)	<0.001
Age groups, *n* (%)	45∼54 years		
55∼64 years		1.94 (1.55, 2.41)	<0.001
65∼74 years		3.22 (2.49, 4.16)	<0.001
≥75 years		4.03 (2.91, 5.57)	<0.001
Education, *n* (%)	> 6 years		
0 years		1.30 (1.00, 1.68)	0.048
1∼6 years		1.16 (0.96, 1.41)	0.136
Hypertension	No	1.85 (1.52, 2.24)	<0.001
Diabetes	No	1.26 (1.02, 1.55)	0.033
Smoking status, *n* (%)	Never smoking		
Passive smoking		1.26 (1.05, 1.53)	0.016
Ever smoking		0.76 (0.50, 1.16)	0.208
Current smoking		1.20 (0.94, 1.55)	0.145
Alcohol consumption, *n* (%)	Never drinking		
Ever drinking		1.09 (0.86, 1.39)	0.468
Current drinking		1.21 (0.52, 1.95)	0.988
LDL-C, mean(SD), mmol/L	—	1.15 (1.08, 1.22)	<0.001

OR, odds ratio; CI, confidence interval; TC, total cholesterol; TG, triglycerides; HDL-C, high density lipoprotein cholesterol; LDL-C, low density lipoprotein cholesterol.

**Table 5:** Multivariate analysis for the risk factors of carotid plaque in this study

Risk factors	References	OR (95% CI)	*P*
Men	Women	1.76 (1.47, 2.11)	<0.001
Age groups, *n* (%)	45∼54 years		
55∼64 years		2.44 (2.03, 2.95)	<0.001
65∼74 years		3.07 (2.43, 3.89)	<0.001
≥75 years		6.39 (4.61, 8.84)	<0.001
Education, *n* (%)	> 6 years		
0 years		1.00 (0.79, 1.27)	0.999
1∼6 years		1.07 (0.90, 1.27)	0.460
Hypertension	No	1.56 (1.32, 1.84)	<0.001
Diabetes	No	1.50 (1.23, 1.84)	<0.001
Smoking status, *n* (%)	Never smoking		
Passive smoking		1.00 (0.84 1.19)	0.992
Ever smoking		0.80 (0.55, 1.17)	0.254
Current smoking		1.23 (0.98, 1.55)	0.079
BMI, mean(SD), Kg/m^2^	—	0.97 (0.95, 0.99)	0.004
LDL-C, mean(SD), mmol/L	—	1.61 (1.51, 1.73)	<0.001

OR, odds ratio; CI, confidence interval; BMI, body mass index; LDL-C, low density lipoprotein cholesterol.

Compared to their references, the prevalence of carotid plaque was increased by 76% in men; 1.44-fold in those aged 55-64 years, 2.07-fold in those aged 65-74 years, and 5.39-fold in those aged ≥ 75 years; 56% in those with hypertension; 50% in those with diabetes; and 61% for 1 mmol/L-increase in LDL-C level. However, carotid plaque prevalence decreased by 3% for each 1 kg/m^2^-increase in BMI (all *P* < 0.0001; Table 4).

## DISCUSSION

This is the first study to evaluate the characteristics of AS based on increased CIMT and carotid plaque in a low-income population in China. Older age, male sex, lower education level, hypertension, diabetes, passive smoking, and high LDL-C level were independent risk factors of early AS. Simultaneously, we found that carotid AS was associated with age, sex, hypertension, diabetes, BMI, and LDL-C level; of these, high BMI was found to be a protective factor, while the remaining variables were all independent risk factors.

CIMT, which may be measured noninvasively by high-resolution ultrasound imaging, has been widely used as an intermediate marker for AS [20]. CIMT has been found to be a biomarker that may reflect the degree of AS [20, 21]. Previous large-scale follow-up studies have shown that elevated CIMT predicts future vascular events independently of conventional vascular risk factors [21–28].

Age and male sex are established risk factors that were significantly associated with increased CIMT [29–31]. Consistent with previous studies, we found that CIMT increased along with age, and there was a higher frequency of increased CIMT in male individuals in this study.

Hypertension, diabetes mellitus, dyslipidemia, and smoking have been shown to be associated with increased CIMT [30–32]. Dyslipidemia has been established to be a dubious effected factor of AS. The levels of TC, TG, HDL-C, and LDL-C were significantly associated with AS in previous reports, and positive relationships were observed between TC, TG, and LDL-C levels [33, 34]; nevertheless, an inverse trend was found for HDL-C level [34]. However, previous studies revealed that TC, TG, and HDL-C levels did not have a significant effect on CIMT [35], and LDL-C level was reported to have an insignificant effect on CIMT [35–37].

In the present study, hypertension and diabetes were independent predictors of elevated CIMT. LDL-C level had an obvious association with increased CIMT; the prevalence was increased by 15% per 1 mmol/L-increase in LDL-C, but there was no association with TG and HDL-C levels.

Recent investigations in large population-based studies have detected equally strong inverse associations between socioeconomic status and preclinical AS assessed by noninvasive ultrasound measurements of the carotid arteries. Previous studies have shown that low education, low income, and manual occupations are associated with CIMT or with faster progression of CIMT [38, 39]. In line with these previous studies, we found an inverse association between education level and CIMT in the multivariate analysis; the risk of increased CIMT decreased by 30% in the illiteracy group compared to the > 6 years education group. Moreover, we found a significant relationship between passive smoking and increased CIMT.

Similar to CIMT, conventional risk factors have been confirmed to be strong risk factors of carotid plaque in previous studies [40, 41]. Age, sex, hypertension, diabetes, TC, and LDL-C were dominating risk factors for developing carotid plaque. Inconsistent with these studies, a protective effect against carotid plaque was observed with increased BMI; the prevalence rate of carotid plaque decreased by 3% with each 1 kg/m^2^-increase in BMI. In white subjects, increased CIMT has been shown to be strongly associated with increased carotid plaque [42], while data from black populations suggest this may not be the case with reduced carotid plaque and large-vessel AS [43]. Black subjects had significantly greater CIMT and a lower prevalence of carotid plaque than white subjects did, after adjusting for cardiovascular risk factors [42, 44]. The discordance of carotid plaque presence and normal CIMT was also observed in other studies [45, 46]. Even when CIMT levels are not elevated, more than 30% of subjects have soft or mixed plaque [47]; of those with CIMT > 1 mm, more than 70% had soft or mixed plaque [48]. Consistent with these studies, we found an inconsistent trend between CIMT and carotid plaque, namely an increased prevalence of carotid plaque and lower mean value of CIMT in this low-income Chinese population. This paradoxical phenomenon can explain in part the high incidence of first-ever stroke in this population.

A report from the Young Finns Study indicated that offspring exposed to parental smoking in childhood had approximately twice the risk of having a carotid AS plaque in adulthood than did those with nonsmoking parents [49]. Carotid IMT in adulthood was greater in those exposed to smoking with both parents than in those whose parents did not smoke [50]. Moreover, in the ARIC study, the larger IMT observed in the nonsmoking group exposed to passive smoking compared with the nonsmoking group not exposed to passive smoking persisted after controlling for diet, physical activity, BMI, alcohol intake, education, and major cardiovascular risk factors [51]. Consistent with the ARIC study, we found that passive smoking was positively associated with increased CIMT in this study, but not with the presence of carotid plaque. The association may be explained by the activation of platelets leading to their recruitment, adherence, and migration to the endothelium [52]; increased oxidative stress resulting in endothelial dysfunction [53]; greater levels of inflammatory markers such as C-reactive protein and oxidized LDL-cholesterol [54]; a weakening of serum antioxidant defense; accelerated lipid peroxidation; and accumulation of LDL-cholesterol in macrophages [55] and reduced levels of HDL-cholesterol [56]. Additional follow-up studies focusing on the progress of carotid plaque are thus needed.

We acknowledge the limitations of this study. First, the study was conducted in a low-income population from a local town in Tianjin, China, and the results may not be representative of the general population. Second, the cross-sectional study design may have led to a selection bias, especially among healthy elderly subjects. However, including only participants with no history of stroke or CVD may have overcome this limitation. Third, there was no information related to grading of carotid AS, and this may have affected the analysis of carotid AS. Moreover, all participants with carotid plaque were asymptomatic. This may have decreased the selection bias. Finally, information regarding medications and measurements of plasma C-reactive protein and homocysteine were absent in the baseline survey, and therefore, we could not assess the relationship between these variables and AS in this study.

In this cross-sectional study, we assessed the prevalence and relevant risk factors of increased CIMT and carotid plaque among participants aged 45 years and over. There was a lower mean CIMT and higher prevalence of carotid plaque in this study across age and gender. Age, sex, hypertension, diabetes, and LDL-C level were significantly associated with CIMT and carotid plaque, but elevated BMI and TC level were protective factors against developing carotid plaque. Therefore, it is vital to identify the factors of AS at different stages to prevent CVD and stroke and to reduce the burden of stroke in this high-risk population.

## MATERIALS AND METHODS

### Study population

This was a population-based cross-sectional study conducted from April 2014 to January 2015.The study population was from the Tianjin Brain Study [4–6]. The study design was described in a previous study [57]. All residents aged 45 years and older with no history of CVD and stroke were recruited in this study, while those with a history of CVD and stroke were excluded.

All investigative protocols were approved by the ethics committee of Tianjin Medical University General Hospital; the methods were carried out in accordance with the approved guidelines, and informed consent was obtained from all participants.

### Epidemiological survey and relevant information

The surveys were conducted through face-to-face interviews by trained research staff to collect name; sex; date of birth; educational level; previous history of hypertension, diabetes mellitus, stroke, transient ischemia, and coronary heart disease; family history of hypertension, diabetes mellitus, stroke, and coronary heart disease; cigarette smoking (≥ 1 cigarette per day for ≥ 1 year); and alcohol consumption (drinking alcohol ≥ 1 time per week for 1 year).

All participants were categorized into four age groups: 45-54 years, 55-64 years, 65-74 years, and ≥ 75 years. Educational level was categorized into three groups according to educational years: illiteracy (no education), 1-6 years, and > 6 years. Individual and family medical histories, which included hypertension (defined as self-reported hypertension, SBP ≥ 140 mmHg, DBP ≥ 90 mmHg, or taking antihypertensive medication), diabetes mellitus (defined as self-reported diabetes, fasting plasma glucose ≥ 7.0 mmol/L, or taking antidiabetic medication), stroke, transient ischemic attack, and coronary heart disease, were obtained according to patient self-reporting or previous records. Lifestyle variables included cigarette smoking, passive smoking (≥ 1 hour/day or exposed to environmental smoking), and alcohol consumption.

### Physical measurements

Physical examinations with measurements of blood pressure (including SBP and DBP), height, and weight were performed; the levels of fasting blood glucose, TC, TG, HDL-C, and LDL-C in serum were measured. BMI was calculated as weight (kg) divided by the square of height (m^2^).

### Ultrasonography measurements

One trained technician blinded to individuals’ previous disease histories performed all ultrasound exams using B-mode ultrasonography (Terason 3000; Burlington, MA, US) with a 5-12 MHz linear array transducer. The CIMT of the far wall of the distal common carotid artery (CCA) was measured as the distance from the leading side of the first echogenic line (lumen-intima interface) to the leading side of the second line (media-adventitia interface). Extracranial carotid artery trees, which included the CCA, the bifurcation, and the internal and external carotid arteries on both sides, were screened for plaque. Examinations included bilateral observation of the longitudinal and transverse views of the CCA. The trained technician performed the carotid ultrasonography with the participants lying in the supine position with the neck extended in mild lateral rotation. The CIMTs for the near and far walls of the CCA were measured both on the left and right, and three values were obtained, which included the maximum CIMT, minimum CIMT, and average CIMT. Images were obtained and digitally stored according to a standard protocol. All scans were recorded on Vascular Research Tools 6 (MIA, LLC) for subsequent off-line analysis. The inter-observer and intra-observer correlation coefficients ranged from 0.88-0.94 and 0.80-0.95 for both sides of the CIMT measurement, respectively.

Carotid plaque was defined as a focal structure encroaching into the arterial lumen by at least 0.5 mm or 50% of the surrounding CIMT value, or a thickness of more than 1.5 mm from the intima-lumen interface to the media adventitia interface [58]. Subjects with carotid plaque were included in the carotid plaque group if they had ≥ 1 identified lesion, regardless of carotid plaque measurements. Both longitudinal and transverse dynamic images of each plaque were stored. Simultaneously, the numbers of carotid plaques and total plaque areas were obtained from each participant with plaque.

All participants were screened by ultrasonographical measurements between April 2014 and July 2014, and all records were analyzed between August 2014 and January 2015.

### AS staging

Increased CIMT was defined as an average CIMT of more than 0.61 mm, which was identified based on the greatest quartile ( < 0.51, 0.51∼, 0.5525∼, and ≥ 0.61 mm). The earlier phase of AS was defined based on the presence of increased CIMT, and the advanced stage of AS was defined based on the presence of carotid plaque.

### Statistical analyses

All subjects were categorized into two groups: the early AS group and advanced AS group. Continuous variables are presented as means and standard deviations and were compared between groups using the Student’s *t*-test. Categorical variables are presented as numbers with frequencies and were compared using chi-square tests or chi-square trend tests. The risk factors of early AS and advanced AS were assessed individually using logistic regression analyses. Factors of early AS were assessed among those without carotid plaque. The univariate analysis results are presented as unadjusted ORs and 95% CIs; the multivariate analysis results are presented as adjusted ORs and 95% CIs after adjusting for covariates. *P* values < 0.05 were considered statistically significant. SPSS for Windows (version 13.0; SPSS Inc., Chicago, IL, USA) was used for analyses.

## Abbreviations

CIMT: carotid intima-media thickness
AS: atherosclerosis
SBP: systolic blood pressure
DBP: diastolic blood pressure
TC: total cholesterol
TG: triglycerides
HDL-C: high-density lipoprotein cholesterol
LDL-C: low-density lipoprotein cholesterol
ORs (95%): odd ratios
CIs: confidence intervals
BMI: body mass index
CVD: cardiovascular disease.
